# The porcine carotid body: morphological and lectin histochemical characterization

**DOI:** 10.3389/fvets.2025.1722075

**Published:** 2026-01-21

**Authors:** Ecaterina Semzenisi, Andrei Ungur, Mihai-Cristian Feher, Alexia-Teodora Hoţa, Dragoṣ Hodor, Romelia Pop, Alexandru-Flaviu Tăbăran

**Affiliations:** 1 Department of Veterinary Pathology, Faculty of Veterinary Medicine, University of Agricultural Sciences and Veterinary Medicine of Cluj-Napoca, Cluj-Napoca, Romania; 2 Department of Porcine Health Management, Faculty of Veterinary Medicine, University of Agricultural Sciences and Veterinary Medicine Cluj-Napoca, Cluj-Napoca, Romania

**Keywords:** carotid body, pig, neuroendocrine, gdnf, IHC, GFAP, S-100, NSE

## Abstract

Although the carotid body (CB) has been widely examined in many animal species, its histological features in pigs have received comparatively little attention. This research presents the first integrative description of the porcine (CB), combining anatomical dissection, histological evaluation (H&E and Masson's trichrome), immunohistochemistry, and lectin histochemistry. In the study, four piglets were examined from which the carotid glomus was harvested, and it was described histologically. The organ displayed a multilobular structure embedded in connective tissue. Immunohistochemical labeling with GFAP, S100, and NSE demonstrated strong and consistent immunoreactivity, confirming the presence of neuroendocrine and glial-like cell populations. Complementary lectin histochemistry with Con-A and WGA revealed distinct glycosylation patterns that correlate with the presence of growth factor receptors (TrkA, TrkB, TrkC, p75NTR, and others), thereby offering insight into receptor biology and glycan-mediated signaling within the (CB).

## 1 Introduction

The (CB) is a tiny, yet vital chemosensory organ, formed from neural crest cells during development. These paired organs are situated on each side of the neck at the point where the common carotid artery splits, perfectly positioned to check the blood before it flows to the brain (1). The porcine carotid artery has long been of interest in studies on hypertension, hypercholesterolemia (2), and atherosclerosis (3), and its anatomy has been well-described in pigs. However, the (CB) remains without significant attention, described sporadically in literature emphasizing more on vascular research, largely due to its obscure location and small size (2, 4–7).

Inside, the CB contains clusters of specialized glomus (type I) cells and supportive sustentacular (type II) cells, surrounded by an intricate network of capillaries and nerve endings. Type I and Type II cells share common progenitor that can generate multiple fates, and under special conditions, some cells have enough plasticity to “reprogram” into another function (8–10).

Clusters of Type I cells contain highly dopaminergic glomus cells that also secrete significant levels of glial cell line–derived neurotrophic factor (GDNF). Given their dual ability to release dopamine and provide sustained neurotrophic support and owing to their unique chemosensory capabilities and remarkable adaptive properties. During the XXI century, glomus cells started to represent a compelling candidate for cell therapy (11, 12). Research has shown increasing promise in treating neurodegenerative disorders such as Parkinson's disease, with favorable results seen in glomus cell auto-transplantation studies involving non-human primates. However, the high cost of these procedures remains a significant barrier, limiting the ability to collect large-scale data (12–14). Certain studies suggest that pigs might be a promising experimental model for (CB) cell transplantation therapy in Parkinson's disease. due to their similar dopaminergic systems. An extensive range of growth factors, along with their corresponding receptors (Table 1), are known to play a role in most plastic modifications of the CB. Additionally, the CB expresses various growth factors, including nerve growth factor (NGF), brain-derived neurotrophic factor (BDNF), glial cell line-derived neurotrophic factor (GDNF), insulin-like growth factors (IGFs), and vascular endothelial growth factor (VEGF). Consequently, CB cells produce dopamine, which PD (Parkinson's disease) patients lack, and also secrete GDNF, which has neuroprotective effects on surviving dopamine neurons. Furthermore, the crosstalk between p75NTR and Trks creates high-affinity NT binding sites on neuronal cells and alters their signaling properties. Understanding their signaling in pigs can provide insights into cell therapy transplantation (8–10, 15).

**Table 1:** Growth factors of CB with their receptors and functions.

**Growth factor**	**Receptor(s)**	**Function**	**Source**
Nerve growth factor (NGF)	TrkA, p75	Neuronal survival, differentiation	(11, 28, 30)
Brain-derived neurotrophic factor (BDNF)	TrkB, p75	Plasticity, neuroprotection	(11, 30, 36)
Neurotrophin-3 (NT-3)	TrkC, p75	Neuronal development, plasticity	(37, 38)
Glial cell line-derived neurotrophic factor (GDNF)	GFRα1, RET, NCAM	Neuroprotection, cell survival	(39, 40)
Neurturin (NRTN), artemin (ARTN), persephin (PSPN)	GFRα2 GFRα3 GFRα4	Neuronal maintenance	(39–42)
Insulin-like growth factor I (IGF-I)	IGF-IR	Growth, survival	(43)

Adapted from STOCCO (15).

The use of pigs in medical research and transplantation is generally considered more ethically acceptable than non-human primates or other species (16, 17). Pigs have shown the best results in cellular therapy for treating hematological malignancies (18).

Porcine (pig-derived) pluripotent chromaffin cells remain a promising source for transplantation (19). Specifically, in humans and mice, the (CB) does not consistently contain chromaffin cells and has an embryological origin different from that of the paraganglionic system. However, in pigs, these cells are consistently present (20). To our knowledge, there are no reported cases in the literature of (CB) histology or detailed descriptions of the CB in pigs.

Since pigs usually don't live beyond the age of 3 years, being either slaughtered for consumption or removed from breeding herds before that, they rarely develop degenerative diseases. This makes such conditions very uncommon in pigs. Although the literature discusses carotid bodies in mice, rats, dogs, and cats, there are no detailed descriptions of the porcine (CB). This may be due to the notable similarities with other species, resulting in a lack of specific histological emphasis.

Although previous work has been done on cell transplantation from the (CB), there is no clear histological description available for pigs. This study aims to provide a comprehensive anatomical, histological, and functional characterization of the porcine carotid body by integrating classical staining with immunohistochemical mapping of glial and neuroendocrine markers (GFAP, S100, NSE), together with lectin histochemistry (Con-A and WGA), to infer the glycoprotein- and receptor-related architecture underlying its neurochemical activity.

## 2 Materials and methods

### 2.1 Animal model

The study involved 16 piglets aged 4–8 weeks from an industrial pig farm in Romania. All animals were of PIC genetic lines, commonly used for meat production. Identifying and preserving the (CB) on both sides proved technically challenging because of its minute size and deep position at the carotid bifurcation. Although the CB is generally located at the posterior aspect of the common carotid artery bifurcation, previous anatomical research has shown that the level, vessel diameter, and branching angles of the left and right carotid arteries often differ due to natural asymmetry of the carotid axis (21). As a result, complete and well-preserved CBs were obtained bilaterally in four animals, which were then selected for detailed histological, immunohistochemical, and lectin-histochemical analyses. The animals were kept under identical environmental conditions and had free access to a balanced diet. Each piglet was clinically examined on the day of the procedure, and individuals showing signs of respiratory distress, dehydration, or fever were excluded. Animals were housed under standard husbandry conditions appropriate for their age, with unrestricted access to water, adequate nutrition, and environmental temperature control.

### 2.2 Ethical approval

All procedures were conducted in accordance with Directive 2010/63/EU on the protection of animals used for scientific purposes and were approved by the institutional ethics committee (Protocol CBE No. 527/03.10.2025). Full protocol of **s**edation, anesthesia, and euthanasia (Supplementary File 1).

### 2.3 Sedation, anesthesia, and euthanasia

#### 2.3.1 Sedation

Piglets received intravenous azaperone (1–2 mg/kg) and ketamine (5–10 mg/kg) via the auricular vein, diluted to 0.5–1.0 ml/kg in sterile saline. Sedation was confirmed by loss of righting reflex, reduced jaw tone, and absence of pedal withdrawal.

#### 2.3.2 Anesthesia

When surgical depth was required, propofol (1–3 mg/kg IV) was administered to effect. Supplemental oxygen was provided by mask.

#### 2.3.3 Euthanasia

Once a deep anesthetic plane was ensured, T-61 (embutramide–mebezonium–tetracaine) was administered intracardially at 0.3 ml/kg. The injection was performed using a 21–22 G, 40–50 mm needle inserted into the left 4th−5th intercostal space. Intracardiac administration was strictly performed only after complete loss of consciousness.

#### 2.3.4 Confirmation of death

Death was confirmed by: absence of corneal reflex, absence of spontaneous respiration, auscultation for ≥5 min, bilateral thoracotomy (final confirmation). All procedural details (drug doses, routes, operator identity) were recorded in the procedural log (Supplementary File 1).

### 2.4 Sample collection and tissue preparation

Necropsies were performed at the Pathology Department of the University of Agricultural Sciences and Veterinary Medicine, Cluj-Napoca, Romania, using standard techniques to locate and isolate the anatomical structures of interest. During the dissection of the region, the common carotid arteries were identified running bilaterally along the trachea. These arteries were traced cranially toward their bifurcation into the internal and external carotid arteries near the base of the skull. The (CB) is a small, oval, reddish-brown structure embedded within the adventitia of the common carotid artery bifurcation that is hardly visible to the naked eye. CB is a small structure. Fine dissection tools were used to separate the (CB) from surrounding connective tissue with minimal damage. The (CB) was excised along with a small portion of the adjacent carotid artery and connective tissue.

### 2.5 Histology

The examination of minute neuroendocrine structures such as the (CB) necessitates meticulous cellular identification due to the intricate composition of neuronal, neuroendocrine, and glial-like cells.

The selected samples were stored in 10% buffered formalin for at least 48 h, routinely processed and embedded in paraffin. Sections of 2–3 μm thick were obtained and stained with hematoxylin-eosin (H&E). Masson's Trichrome staining was performed according to the standardized protocol (22).

### 2.6 Immunohistochemistry

Immunohistochemical labeling employing S100, NSE, and GFAP provides a dependable and insightful methodology for distinguishing and mapping these cellular populations, thereby facilitating a comprehensive understanding of the tissues' architecture and functional attributes.

Immunohistochemistry (IHC) was performed for Glial Fibrillary Acidic Protein (GFAP; mouse monoclonal, clone GA5, Leica Biosystems, REF PA0026), Neuron-Specific Enolase (NSE; mouse monoclonal, clone 22C9, Leica Biosystems, REF PA0435), and S100 protein (rabbit polyclonal, Leica Biosystems, REF PA0900). All slides were processed automatically using the Leica Bond-Max™ immunostaining system (Leica Biosystems, Melbourne; model M2 12154). Detection was performed with the Bond Polymer Refine Detection Kit (Leica Biosystems, DS9800), which incorporates a Post-Primary Rabbit anti-Mouse IgG reagent and an HRP-polymer complex. The signal was visualized with 3,3′-diaminobenzidine (DAB), followed by counterstaining with Mayer's hematoxylin and mounting in DPX medium. GFAP and NSE showed brown cytoplasmic staining, whereas S100 exhibited both cytoplasmic and nuclear positivity. Appropriate positive controls were included for each marker, and negative controls were obtained by omitting the primary antibody.

Immunoreactivity was semi-quantitatively assessed using an H-score, originally described by McCarty et al. (23) in which all nucleated cells within representative high-power fields (400 × ) were evaluated and assigned a staining intensity of negative (0), weak (1+), moderate (2+), or strong (3+). The H-score for each marker was calculated using the standard formula—(1 × %1+) + (2 × %2+) + (3 × %3+)—yielding a theoretical range from 0 to 300. This method has been widely applied for the quantitative evaluation of immunohistochemical expression in tissue sections.

### 2.7 Lectin histochemistry

Because of their high specificity for cellular structures and their ability to map glycoprotein trafficking or identify active secretion zones in endocrine and neuroendocrine cells, it is reasonable to use lectin histochemistry as a method for detecting specific cellular components (24, 25). The major neurotrophic factors and their receptors previously identified in (CB) tissuewere summarized in Table 2. Because many of these receptors are glycosylated, they provide a biochemical rationale for the use of Con-A and WGA in this study, allowing indirect visualization of receptor-associated glycan domains.

**Table 2:** Mapping lectins to growth factor receptors from pig CB carotid body (based on glycosylation).

**Growth factor**	**Receptor**	**Glycosylation type**	**Lectins**	**Binding sites**	**Source**
Nerve growth factor (NGF)	TrkA, p75	N-linked (mannose/glucose rich)	Con-A, WGA	Con-A bindsα-mannose/glucose on TrkA; WGA binds GlcNac on p75.	(26, 27)
Brain-derived neurotrophic factor (BDNF)	TrkB, p75	N-linked, sialylated	WGA, SNA,	WGA for GlcNAc; SNA/MAL-I for sialic acid types	(28)
NT-3	TrkC, p75	N-linked, sialylated	WGA, SNA	TrkC is highly glycosylated; WGA for GlcNAc, SNA for α2,6 sialic acid	(28)
Glial cell line-derived neurotrophic factor (GDNF).	GFRα1, RET, NCAM	Highly glycosylated (mannose/GlcNAc-rich)	Con-A, WGA	GDNF receptors are rich in N-linked sugars	(26, 27)
NRTN, ARTN, PSPN	GFRα2/3/4	GPI-anchored, glycosylated	WGA, Con-A, possibly PHA-L	To reveal complex N-glycans and GlcNAc	(44, 45)
IGF-I	IGF-IR	Complex N-glycans	PHA-L, Con-A, ECL	PHA-L detects bisected GlcNAc; ECL binds Galβ1-4GlcNAc	(45)

Concanavalin A (Con-A) binds explicitly to α-mannose and α-glucose residues (monosaccharide units or simple sugars). Therefore, it is biochemically possible and that Con-A binds to TrkA via its N-linked mannose or glucoses glycans during glycoprotein profiling assays (26, 27). In addition, there are several lectin types, such as Wheat Germ Agglutinin (WGA), which bind to terminal N-acetylglucosamine (GlcNAc), a common component of N-linked glycans.

The study carried out by Gong et al. (28) uses a rat–human hybrid system for crystallography to investigate the interaction between neurotrophins and p75NTR. In conclusion, Con-A, WGA, and SNA specifically target essential glycan residues (GlcNAc and sialic acid) that are known to be present on TrkB, p75NTR, and other growth factors. This lectin allows accurate and functionally significant analysis of receptor glycosylation, which is essential for understanding receptor biology, trafficking, and ligand interaction in neuronal systems.

For lectin histochemistry, the slides containing the bifurcation of the common carotid artery (CCA) were routinely processed and incubated at room temperature for 60 min with Concanavalin A (Con A; Vector Laboratories, Rhodamine Lectin Kit, RLK-2200) and Sambucus nigra agglutinin (SNA; Vector Laboratories, Rhodamine Lectin Kit, RLK-2200). After incubation, the samples were rinsed twice in distilled water and counterstained with Draq5 for 5 min to visualize. The staining procedures were performed according to the manufacturer's protocols.

### 2.8 Confocal microscopy and image analysis

Confocal fluorescent images were captured with a Zeiss LSM 710 laser scanning system attached to an Axio Observer Z1 inverted microscope. Cell structures were visualized using 543 and 633 nm excitation laser lines to detect Rhodamine (emission BP 548–629 nm) and Draq5 (emission BP 661–759 nm), respectively. Images were collected with a Plan-Apochromat 63 × /1.4 oil immersion objective (DIC 27). The beam pathway was split with an MBS 488/543/633 filter. Image acquisition, processing, and analysis were carried out using the ZEN software package (Zeiss). Fluorescence intensity values are presented as arbitrary units (AU), following previously established protocols (29).

## 3 Results

**Figure 1:**
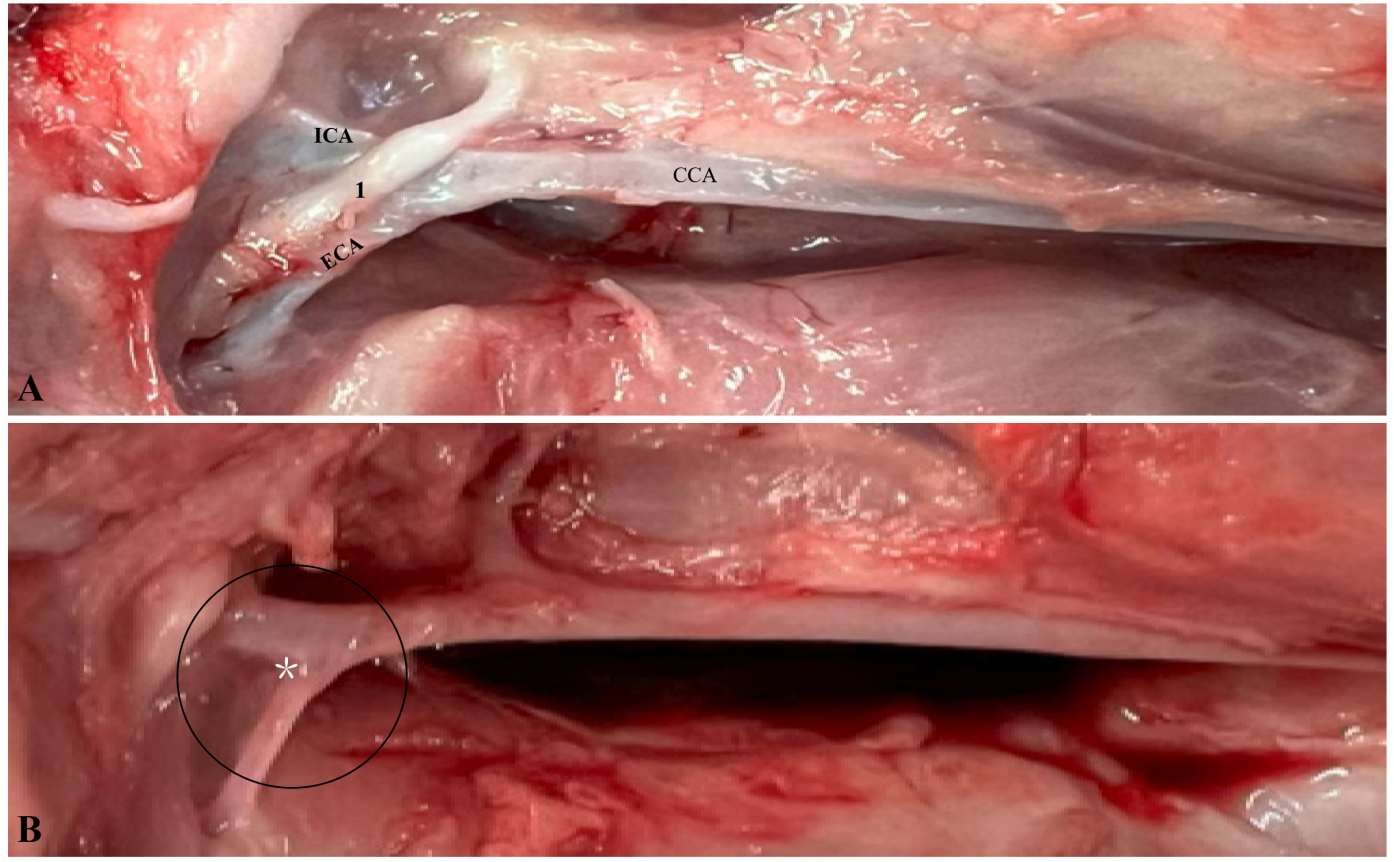
Swine carotid bifurcation: intact and denervated anatomy. **(A)** The common carotid artery (CCA) dividing into the internal carotid artery (ICA) and external carotid artery (ECA). The vagus nerve (1) is visible, overlying the bifurcation. **(B)** The denervated CCA, with the bifurcation encircled and the carotid body (CB) marked by a white asterisk.

Grossly, the (CB) is a small, ovoid structure located at the bifurcation of the common carotid artery, where the vessel divides into the internal and external carotid arteries. On gross inspection, it presents as a soft, reddish-brown nodule; however, its minute size and close integration within the vascular wall make it difficult to identify during routine dissection. Anatomically, it is located in proximity to the vagus nerve and receives consistent innervation from the carotid sinus nerve, a branch of the glossopharyngeal nerve (cranial nerve IX), which extends into a fine plexus enveloping both the carotid sinus and the (CB; Figure 1).

**Figure 2:**
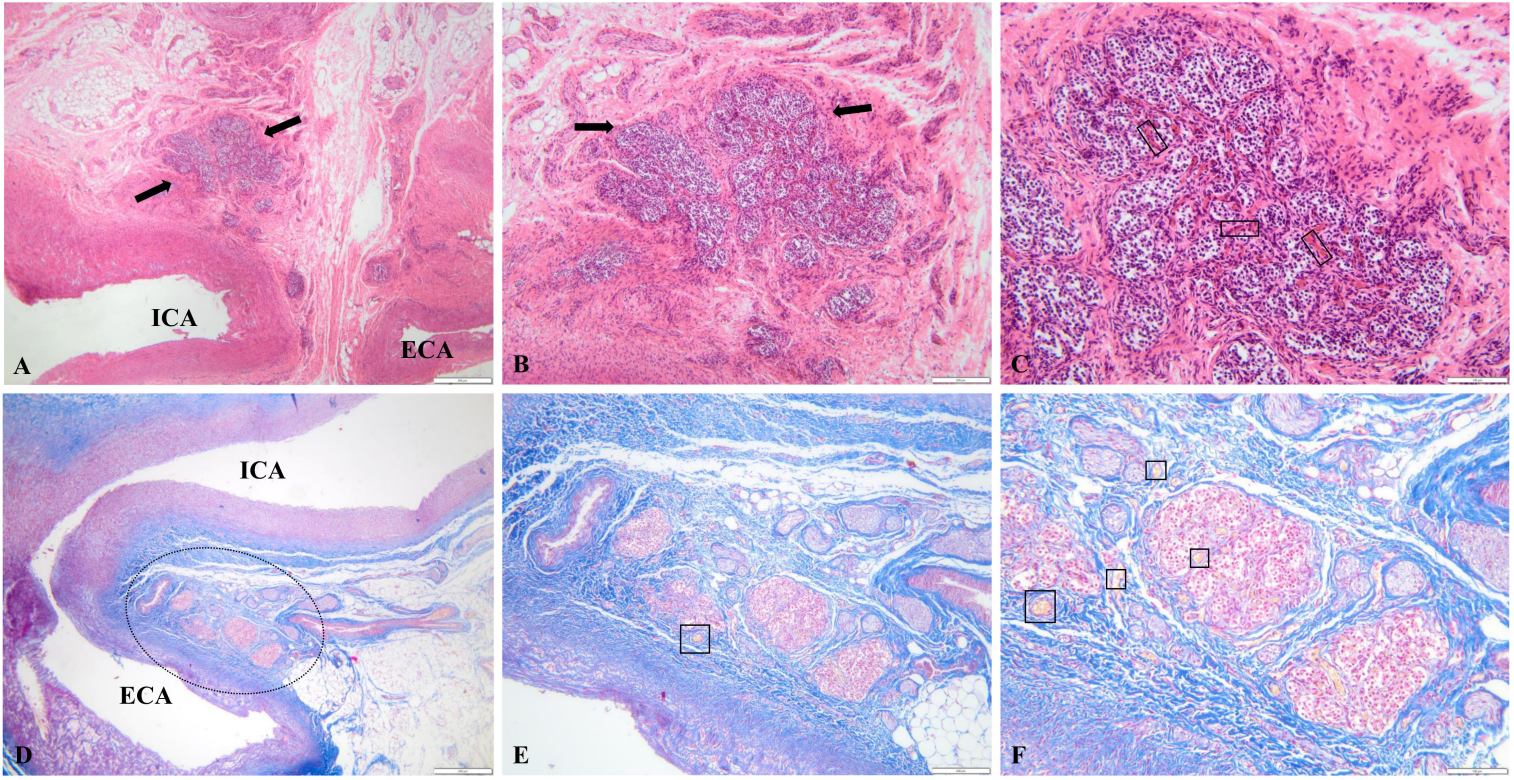
Histological features of the carotid body. **(A–C)** Anatomical relationship between the carotid body (CB), internal carotid artery (ICA), and external carotid artery (ECA). The black arrows and dashed circle indicate the CB with its characteristic multilobular architecture. In panel C, polygonal Type I cells and elongated Type II sustentacular cells are visible within the lobules, surrounded by a dense capillary network (black rectangles). In panel E and F, black squares delineate the peripheral nerve bundles associated with the CB (H&E; bars = 100, 50, 20 μm). **(D–F)** Connective tissue surrounding the multilobular structure of the CB (Masson's Trichrome; bars = 100, 50, 20 μm).

Histological examination of the porcine (CB) revealed a lobular organization composed of well-defined clusters (glomeruli) separated by thin connective tissue septa (Figure 2). Each cluster was closely associated with a dense capillary network. Within the lobules, two main cell types were identified. Type I (glomus) cells were round to ovoid, centrally located, and contained dense-core vesicles, consistent with their neuroendocrine function. Type II (sustentacular) cells were spindle-shaped, positioned at the periphery of the clusters, and exhibited glial-like morphology. The stromal framework consisted of fibrous connective tissue traversed by numerous nerve fibers. The overall structure reflected the highly vascularized and innervated nature of the organ, consistent with its role as a peripheral chemoreceptor.

NSE immunoreactivity was moderate to strong within glomus cell clusters. The H-score was 205, reflecting the predominance of 2+ and 3+ cytoplasmic staining across the examined fields.

S100 immunoreactivity predominantly involved sustentacular cells at the periphery of CB lobules, with moderate-to-strong cytoplasmic positivity. The calculated H-score was 175, based on 20% strong (3+), 45% moderate (2+), and 25% weak (1+) staining.

GFAP staining showed weak-to-moderate cytoplasmic positivity in scattered sustentacular cells, with a calculated H-score of 130 (10% strong, 30% moderate, 40% weak).

**Figure 3:**
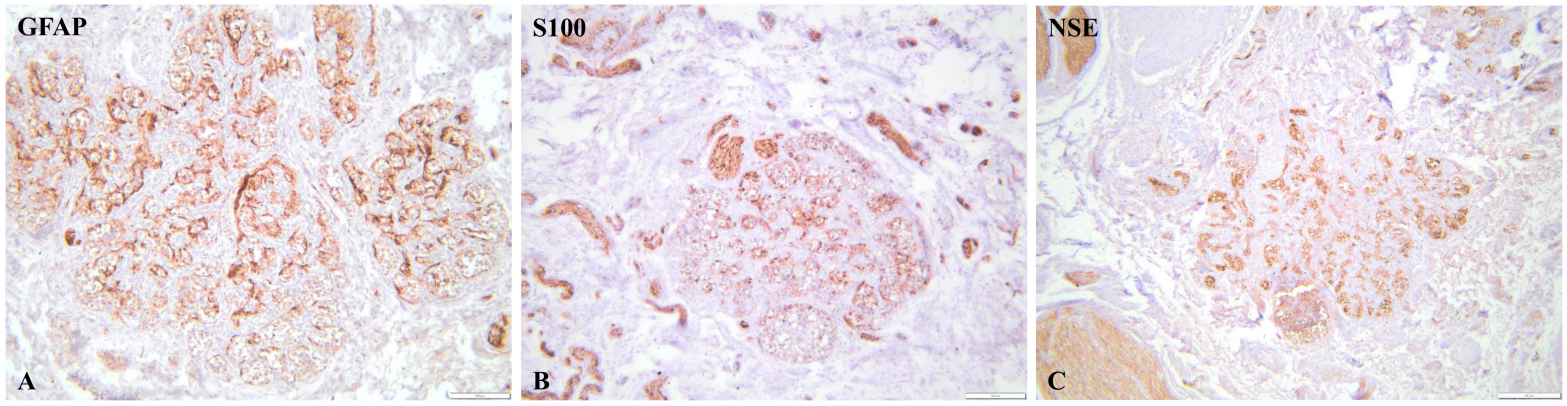
Immunohistochemical features of the carotid body. **(A, C)** Glial markers showed a strong to diffuse immunoreactivity of the sustentacular (type II) cells distributed around the glomeruli (GFAP, NSE, bar = 100 μm). **(B)** Neuronal marker showed a strong diffuse cytoplasmic immunolabelling in type I (glomus) cells. (NSE, bar = 100 μm).

NSE showed strong and diffuse cytoplasmic immunoreactivity (H-score 205), while S100 (H-score 175) and GFAP (H-score 130) highlighted the sustentacular cell framework, confirming the dual neuroendocrine and glial-like cellular composition of the carotid body (Figure 3, Table 3). Representative micrographs with corresponding positive and negative controls are provided in Supplementary File 2.

**Table 3:** Semi-quantitative immunohistochemical evaluation (H-score analysis).

**Marker**	**Predominant cell type**	**Staining pattern**	**Intensity distribution**	**H-score**
NSE	Glomus (type I) cells	Strong, diffuse cytoplasmic	5% weak (1+), 35% moderate (2+), 60% strong (3+)	205
S100	Sustentacular (type II) cells	Moderate–strong cytoplasmic	25% weak (1+), 45% moderate (2+), 20% strong (3+)	175
GFAP	Sustentacular (type II) cells	Weak–moderate cytoplasmic	40% weak (1+), 30% moderate (2+), 10% strong (3+)	130

**Figure 4:**
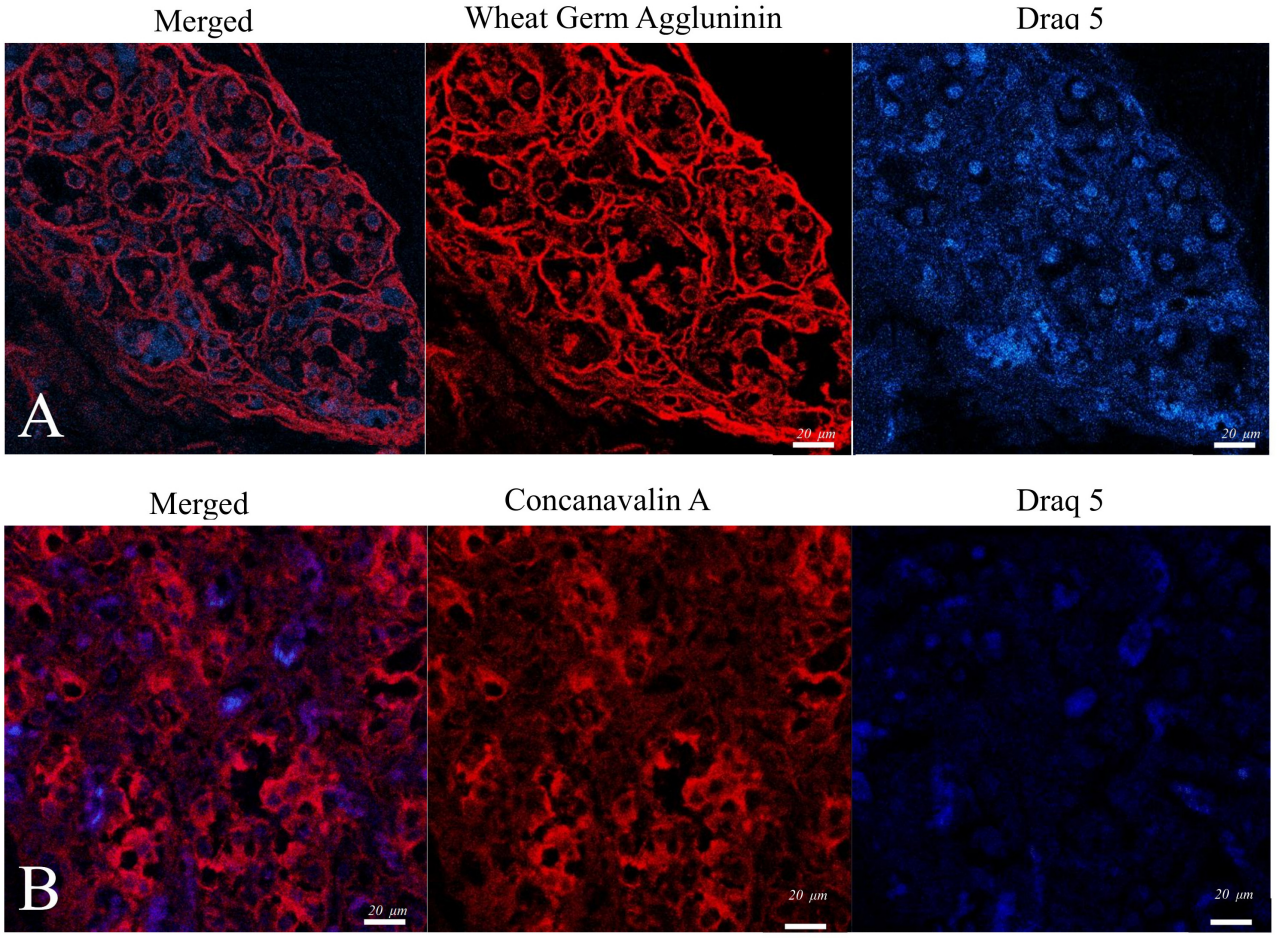
Confocal microscopy micrographs of the CB tissue of a pig. **(A)**. Perinuclear and cell membrane expression of Con-A. **(B)** WGA -showed intense labeling of cell membranes.

Complementary lectin histochemistry using Con-A and SNA (Figure 4) demonstrated distinct glycosylation profiles that align with glycan motifs previously described on neurotrophin receptor families (TrkA, TrkB, TrkC, and p75NTR). These associations are outlined in Table 2, which summarizes the established lectin–glycan binding specificities reported in the literature.

## 4 Discussions

This work offers the first detailed description of the porcine carotid body (CB) from both a morphological and lectin-histochemical perspective. Our findings show that the CB in pigs has the familiar multilobular structure seen in other mammals. Each lobule is composed of compact nests of Type I (glomus) cells surrounded by Type II (sustentacular) glial-like cells, all embedded in a rich network of capillaries. This arrangement underlines the CB's role as a highly vascularized chemoreceptor, specialized in sensing oxygen and carbon-dioxide fluctuations—an organization very similar to that described in dogs, rats, and humans (9, 10).

The immunohistochemical staining pattern we observed with GFAP, S100, and NSE was strong and diffuse, pointing to a close coexistence of glial and neuroendocrine elements. This profile mirrors earlier observations in other species, where S100 and GFAP highlight sustentacular cells and NSE labels metabolically active glomus cells (11, 30). The intensity of the signal in our material could also reflect the young age of the animals; at 4–8 weeks, glial differentiation and synaptic maturation are still highly active processes. A similar pattern has been described in developing rodents (9).

The lectin staining added another layer of information. Concanavalin A (Con-A) and Sambucus nigra agglutinin (SNA) showed complementary binding profiles within the tissue. Con-A, which recognizes α-mannose and α-glucose residues, gave a strong perinuclear and cytoplasmic reaction, suggesting a high content of N-linked glycoproteins involved in receptor trafficking and secretion. SNA, on the other hand, binds terminal sialic-acid residues and labeled mainly the cell membranes, consistent with sialylated receptors such as TrkB, TrkC, and p75NTR, which are active in neurotrophic signaling (15, 28). These differences likely point to cell-specific glycosylation patterns that shape receptor behavior and ligand interactions—something suggested in rodents but demonstrated here for the first time in pigs.

Overall, the data indicate that the porcine CB is not only structurally distinctive but also functionally comparable to the CB of other mammals. Pigs bring several translational advantages over small laboratory animals: their cardiovascular system and metabolic physiology resemble those of humans, they are widely accepted for biomedical use, and they have already proven valuable in xenotransplantation research (16, 31). The consistent neuroglial and glycan expression patterns we observed strengthen the idea that the porcine CB could serve as a useful model for studying neuroregeneration and for developing dopaminergic or glial-based cell therapies for Parkinson's disease.

Recent advances in genetic engineering make this prospect even more relevant. Introducing human complement- and coagulation-regulatory genes has already allowed long-term survival of pig organs in non-human primates, bringing clinical xenotransplantation closer to reality (31). Tools such as whole-genome sequencing, CRISPR editing, and modern proteomic approaches can now be used to refine and characterize porcine CB cells with great precision. When combined with reproductive technologies like cloning, they can yield standardized, genetically defined donor lines suitable for translational experiments (16, 32).

The medical context also adds urgency. The worldwide demand for transplantable tissues still far exceeds the available supply. In Europe and globally, less than one tenth of the need is met, and thousands of patients die each year while waiting for organs (33, 34). At the same time, the prevalence of Parkinson's disease has more than doubled in the last two decades, now affecting over 8.5 million people worldwide (35). These trends make it clear that new, ethically acceptable cellular sources are needed. In this light, the porcine CB with its population of dopaminergic and glial-like cells emerges as a realistic and promising option for future neuroregenerative and xenotransplantation research.

### 4.1 Limitations of the study

This study has a few important limitations to acknowledge. The number of animals was relatively small, although sixteen piglets were initially examined, complete bilateral carotid bodies were successfully collected and analyzed from only four. Still, given that this is the first detailed description of the porcine carotid body, these samples provided a solid and representative foundation for morphological and histochemical characterization. Because of the organ's minute size and deep vascular position, slight sampling bias or partial loss of tissue during dissection cannot be entirely ruled out. The study also focused on young piglets of a single genetic line, which may not capture age- or breed-related variations. Finally, the immunohistochemical and lectin panels were intentionally limited to key markers, and functional aspects such as oxygen sensitivity or receptor signaling were beyond the scope of this work. These aspects should be explored in future studies to expand the biological and translational relevance of our findings.

## 5 Conclusions

This work provides the first integrative description of the porcine (CB), combining anatomical dissection, histological evaluation (H&E and Masson's trichrome), immunohistochemistry, and lectin histochemistry. To date, no such comprehensive characterization of the pig (CB) has been reported. In the context of 21st-century biomedical research, where comparative and translational models are increasingly valuable, this integrative description provides a novel and essential baseline for future investigations into the physiology and pathology of this chemoreceptive organ.

## Data Availability

The raw data supporting the conclusions of this article will be made available by the authors, without undue reservation.
