# Supplementary material for: The porcine carotid body: morphological and lectin histochemical characterization
Source: Front Vet Sci. 2026 Jan 21;12:1722075. doi: 10.3389/fvets.2025.1722075 (PMC12867842; doi:10.3389/fvets.2025.1722075)
Supplement: Supplementary file 1 [file Data_Sheet_1.pdf]

Full protocol.

2.3 Sedation, Anesthesia, and Euthanasia

Sedation and analgesia (intravenous):
Piglets were sedated with an intravenous combination of azaperone (1–2 mg/kg) and ketamine (5–10 mg/kg), administered slowly via the auricular vein. The total volume was diluted in sterile saline to 0.5–1.0 mL/kg to minimize vascular irritation. Within 3–5 minutes, the animals became recumbent and relaxed; they were then placed in sternal position on a warmed surface to maintain normothermia. Adequate sedation was confirmed by loss of the righting reflex, reduced jaw tone, and absence of a withdrawal response to firm digital pressure at the coronary band.

Anesthetic plane prior to euthanasia:
When required to achieve a surgical depth of anesthesia, propofol (1–3 mg/kg IV, to effect) was titrated until palpebral and pedal reflexes were absent and spontaneous respiration became slow and regular. Supplemental oxygen was provided by mask throughout the procedure to ensure adequate oxygenation.

Euthanasia.
Once a deep, stable plane of anesthesia was confirmed, embutramide–mebezonium–tetracaine (T-61) was administered intracardially at a dose of 0.3 mL/kg. The injection site was located at the left 4th–5th intercostal space near the costochondral junction. A 21–22 G, 40–50 mm needle was directed toward the opposite shoulder until ventricular blood flashback was observed, after which the solution was injected slowly in accordance with the manufacturer's instructions. Intracardiac administration was performed only after complete loss of consciousness to ensure that the animals experienced no perception of pain or distress.

Confirmation of death:
Cessation of corneal reflex and spontaneous respiration was verified, followed by auscultation for at least five minutes to confirm cardiac arrest. Pupils were fixed and dilated. As an additional safeguard, a bilateral thoracotomy was performed to confirm death before necropsy or tissue sampling. For each animal, all relevant parameters—including drug doses, administration routes, operator identity, and confirmation criteria—were recorded in the procedural log.

Necropsies were performed at the Pathology Department of the University of Agricultural Sciences and Veterinary Medicine, Cluj-Napoca, Romania, using standard techniques to locate and isolate the anatomical structures of interest.
