# Supplementary material for: The porcine carotid body: morphological and lectin histochemical characterization
Source: Front Vet Sci. 2026 Jan 21;12:1722075. doi: 10.3389/fvets.2025.1722075 (PMC12867842; doi:10.3389/fvets.2025.1722075)
Supplement: Supplementary file 2 [file Data_Sheet_2.pdf]

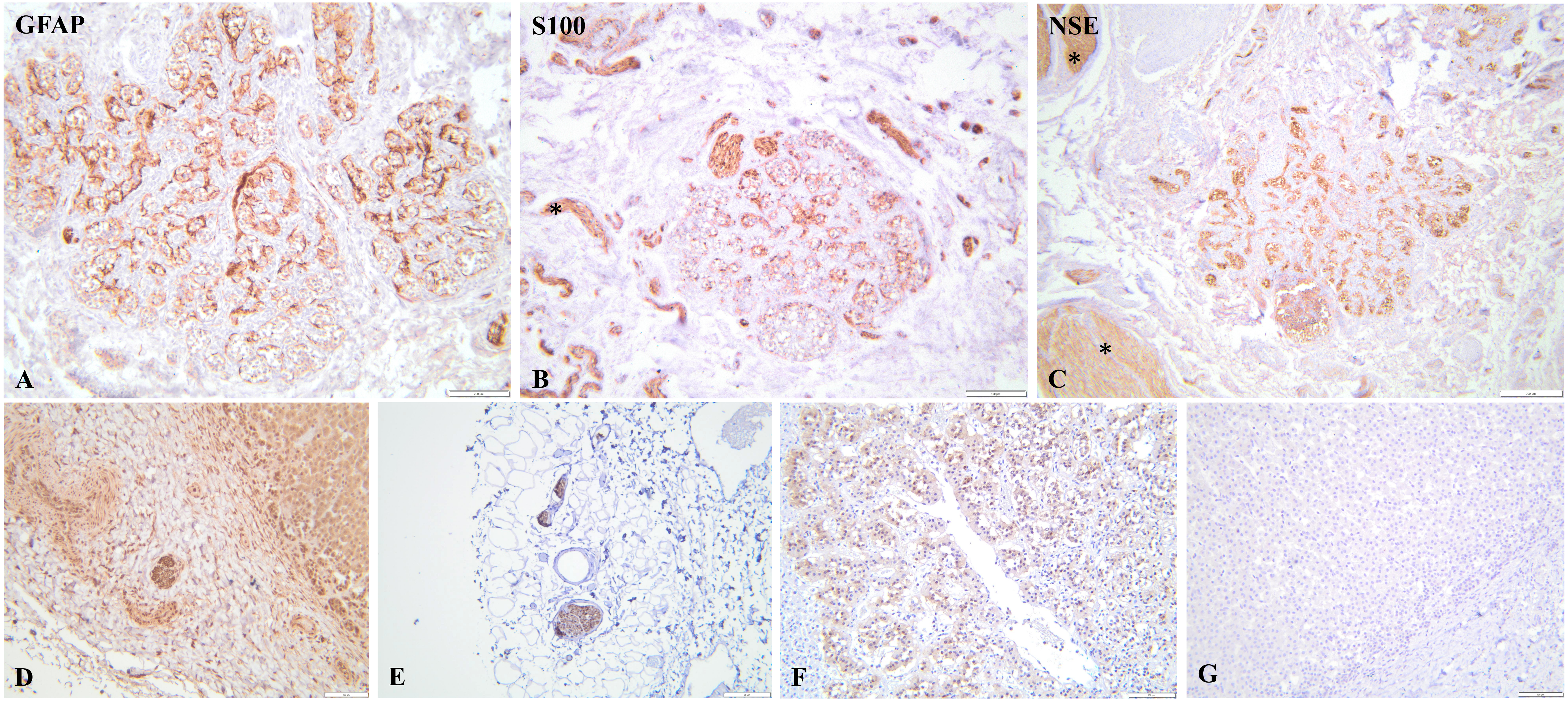

Immunohistochemical expression of glial and neuroendocrine markers in the porcine carotid body.
(A–C) Carotid body sections showing strong cytoplasmic immunoreactivity for GFAP (A), S100 (B), and NSE (C) within glomerular clusters.
(D–F) Corresponding positive controls demonstrating expected expression patterns for GFAP (D), S100 (E), and NSE (F). In panel B and C, internal positive elements (nerve fibers) marked with asterics (*) within the same section further validate antigen preservation and staining specificity.
(G) Negative control processed without primary antibody showing no specific signal.
